# Anticancer Effect of Fucoidan on DU-145 Prostate Cancer Cells through Inhibition of PI3K/Akt and MAPK Pathway Expression

**DOI:** 10.3390/md14070126

**Published:** 2016-07-07

**Authors:** Gang-Sik Choo, Hae-Nim Lee, Seong-Ah Shin, Hyeong-Jin Kim, Ji-Youn Jung

**Affiliations:** Department of Companion and Laboratory Animal Science, Kongju National University, Yesan 340-702, Korea; chu_0602@naver.com (G.-S.C.); lhn2726@naver.com(H.-N.L.); shinsaya@naver.com(S.-A.S.); tigershout@kongju.ac.kr(H.-J.K.)

**Keywords:** apoptosis, fucoidan, human prostate cancer, phosphoinositide 3-kinase, Akt, mitogen-activated protein kinases

## Abstract

In this study, we showed that PI3K/Akt signaling mediates fucoidan’s anticancer effects on prostate cancer cells, including suppression of proliferation. Fucoidan significantly decreased viability of DU-145 cancer cells in a concentration-dependent manner as shown by MTT [3-(4,5-dimethylthiazol-2-yl)-2,5-diphenyltetrazolium bromide] assay. The drug also significantly increased chromatin condensation, which indicates apoptosis, in a concentration-dependent manner as shown by DAPI (4′,6-diamidino-2-phenylindole) staining. Fucoidan increased expression of Bax, cleaved poly-ADP ribose polymerase and cleaved caspase-9, and decreased of the Bcl-2, p-Akt, p-PI3K, p-P38, and p-ERK in a concentration-dependent manner. In vivo*,* fucoidan (at 5 and 10 mg/kg) significantly decreased tumor volume, and increased apoptosis as assessed by the TUNEL (terminal deoxynucleotidyl transferase dUTP nick end labeling) assay, confirming the tumor inhibitory effect. The drug also increased expression of p-Akt and p-ERK as shown by immunohistochemistry staining. Therefore, fucoidan may be a promising cancer preventive medicine due to its growth inhibitory effects and induction of apoptosis in human prostate cancer cells.

## 1. Introduction

Cancer is a life-threatening disease that occurs worldwide; in particular, prostate cancer is the most common cancer affecting men in America and is the second leading cause of cancer-related death [1]. Its incidence and mortality in Korea has also increased because of westernized eating habits and an aging population [2,3]. Prostate cancer is initially androgen-dependent and is limited to local or regional stages for which androgen-ablative therapy is applied to suppress the cancer, but in most cases it progresses to androgen-independent disease [4,5]. Complementary and alternative medicine is used to treat prostate cancer by exploiting various nutritional products, in addition to drugs and dietary supplements [6]. Fucoidan is extremely sticky and it has a high molecular weight. Also, it is a sulfated polysaccharide found in the cell wall matrix of brown seaweeds, such as *Undaria pinnatifida*, *Laminaria angustata*, *Fucus vesiculosus*, and *Fucus evanescens* [7]. Fucoidan is water-soluble polysaccharides having sulfuric acid groups and consist of d-galactose, d-mannose, d-xylose, d-fucose, and sulfate being structurally divided into galacto fucan sulfate. Fucoidan was reported to induce cell death through apoptosis in colon, breast, and liver cancer cells and also inhibits cancer cell growth by blocking cell cycle progression [8,9,10,11,12]. Furthermore, fucoidan’s anti-inflammatory [13], antiviral [14] and anticoagulant [15] effects have received much attention.

More recent studies indicate that chemotherapies based on naturally available marine seaweeds suppress the pathways of mitogen-activated protein kinases (MAPK) and phosphoinositide 3-kinase/protein kinase B (PI3K/Akt) [16,17]. According to the previous study, fucoidan induced apoptosis by the inactivation of p38 MAPK and PI3K/Akt in the PC-3 human prostate cancer cells [18].

MAPK signaling is divided into three subtypes, ERK (extracellular signal-regulated protein kinase), P38 MAPK, and JNK/SAPK (c-Jun *N*-terminal kinase/stress-activated protein kinase), and is known to play a key role in modulating bioactivities such as intracellular responses [19]. ERK, which comprises two isoenzymes, p44^ERK1^ and p42^ERK2^, was the first MAPK to be discovered; when ERKs receive the signal from Ras present in the plasma membrane, they are translocated from the cytoplasm to the nucleus and activate c-Fos and c-Jun and induce the activation of cyclin D1, involved in the cell cycle. Such ERKs are known to be significantly involved in the regulation of various cellular responses, including cell division, proliferation, differentiation, and survival [20].

Akt, also called protein kinase B, is a serine/threonine kinase that was first discovered as a viral oncogene (v-Akt) and exists as three types—Akt1 (PKBα), Akt2 (PKBβ), and Akt3 (PKBγ)—that plays a key role in cell growth and survival [21]. Akt is activated by phosphoinositide 3-kinase (PI3K) and is involved in many processes of intracellular signal transduction such as cell proliferation, differentiation, and angiopoiesis through phosphorylation and activation in the cell membrane [22]. A high level of Akt activation has been found in many cancer cells, including breast and prostate cancer cells, and mutations in Akt or alterations in the activating mechanism of PI3K drive cancer cell growth and resistance to apoptosis, thus acting as a key factor that initiates cancer [23]. Experiments both in vitro and in vivo have validated that the suppression of Akt signaling inhibits proliferation of cancer cells and induces apoptosis; therefore, blocking the Akt signaling pathway can serve to inhibit the abnormal proliferation and growth of tumor cells [24].

In this study, in vitro experiments were performed using androgen-independent DU-145 human prostate carcinoma cells to verify whether fucoidan is effective in inducing apoptosis and has an effect on MAPK and PI3K/Akt signaling. In addition, in vivo experiments to establish the apoptotic effect of fucoidan in prostate cancer has not been reported yet, so we performed in vivo experiment to determine whether fucoidan suppresses tumor growth.

## 2. Results and Discussion

### 2.1. Effect of Fucoidan on the Viability of DU-145 Cancer Cells

**Figure 1:**
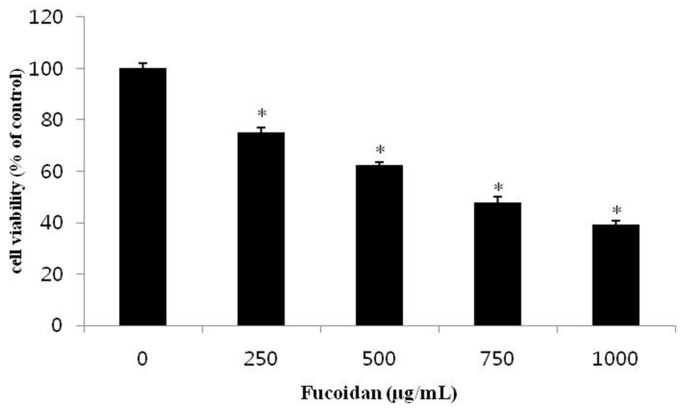
Effect of fucoidan on the cell viability of DU-145 cells. DU-145 cells (2 × 10^4^ cells/mL) were treated with 0, 250, 500, 750, 1000 μg/mL fucoidan in RPMI-1640 medium containing 5% FBS for 24 h. The growth inhibition was measured by the MTT assay. Data are mean standard deviation (SD) for three samples. The significance was determined by Student’s *t*-test (* *p* < 0.05 compared with untreated control).

In this study, the survival rate of cancer cells was measured by the MTT [3-(4,5-dimethylthiazol-2-yl)-2,5-diphenyltetrazolium bromide] assay to explore the effect of fucoidan against DU-145 prostate cancer cells lines. When DU-145 cells were treated with 0, 250, 500, 750, or 1000 µg/mL fucoidan for 24 h, the survival rates of the DU-145 cells were 75.1%, 62.2%, 47.7%, and 39.1%, respectively, all significantly reduced compared with the control group (Figure 1). Boo et al. [18] also reported similar result that after treatment of focoidan, the cell viability of PC-3 prostate cancer cells were significantly reduced in dose dependent manner. Yamasaki et al. [11] reported that the survival rate of MCF-7 breast cancer cells was 25.0% following 1000 µg/mL fucoidan treatment for 24 h, while Yang et al. [12] found a similar effect, with 24 h fucoidan treatment decreasing survival of SMMC-7721 liver cancer cells in a concentration-dependent manner to 40.0% at 500 µg/mL fucoidan. The present results are in agreement with these, though inhibition of survival was slightly less at the same concentrations of fucoidan. Thus, fucoidan suppresses the proliferation of DU-145 prostate cancer cells in a concentration-dependent manner.

### 2.2. The Morphological Changes of DU-145 Cancer Cells by the Fucoidan 

**Figure 2:**
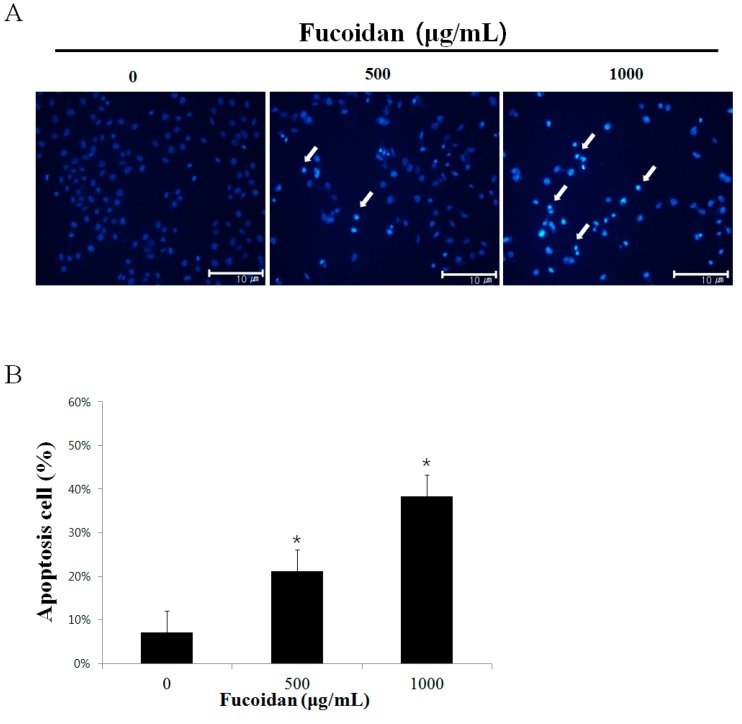
Effect of fucoidan on the chromatin condensation in DU-145 cells. (**A**) DU-145 cells were treated with 0, 500, 1000 μg/mL fucoidan or vehicle in RPMI-1640 medium containing 5% FBS for 24 h, and cell were stained with DAPI. The arrows indicate chromatin condensation in the cancer cell. (**B**) DU-145 cells were treated with fucoidan (0, 500, 1000 μg/mL) for 24 h. Apoptosis cells were counted under a light microscope and expressed as the average of five fields. Each bar represents the mean ± SD calculated from independent experiments. Significance was determined by Dunnett’s *t*-test with * *p* < 0.05 compared as statistically significant compared with non-treated controls.

To investigate whether fucoidan’s suppression of DU-145 cells proliferation is attributable to apoptosis, DU-145 prostate cancer cells were treated with 500 or 1000 µg/mL fucoidan, and then chromosomal condensation was observed by DAPI (4′,6-diamidino-2-phenylindole) staining and fluorescence microscopy (Figure 2A). Increased apoptosis elicited by the cells was observed in the fucoidan-treated group, and, consistent with the MTT assay, DAPI staining showed a reduction in the number of cancer cells in the fucoidan-treated group compared with the control group. Furthermore, fucoidan increased cytoplasmic shrinkage and apoptotic body formation compared with the control group by 7.0 ± 1.5%, 22.4 ± 1.2%, and 36.0 ± 1.3% at 0, 500, and 1000 µg/mL fucoidan, respectively (Figure 2B). These rates were calculated by evaluating of 100 cells by fluorescence microscopy at a magnification of ×200 after selecting five regions randomly. According to the study by Park et al. [23], apoptosis was observed after AGS gastric cancer cells were treated with 100 µg/mL fucoidan; a higher concentration of fucoidan yielded a greater apoptosis effect. These results suggest that DU-145 prostate cancer cells were killed by fucoidan by induction of apoptosis.

### 2.3. Effect of Fucoidan on the Apoptosis-Related Proteins of DU-145 Cancer Cells

**Figure 3:**
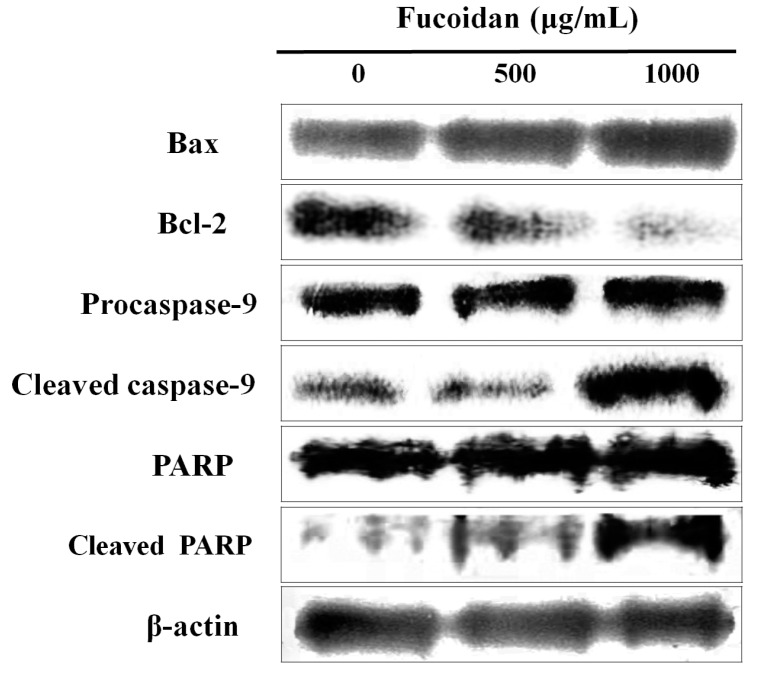
Effect of fucoidan on the apoptotic pathway in DU-145 cells. DU-145 cells were treated with fucoidan 0, 500, and 1000 μg/mL for 24 h and cell were harvested to measure protein levels of Bax, Bcl-2, cspase-9, and PARP by western blotting. The blots were also probed with β-actin antibodies to confirm equal sample loading.

The Bcl-2 family, which comprises proteins that change mitochondrial membrane permeability, plays a key role in regulating apoptosis [24]. Among the Bcl-2 family proteins, Bcl-2 hinders apoptosis, whereas Bax promotes it [25]. When these two signals are out of balance, Bax releases cytochrome c by changing the electric potential of the mitochondrial membrane, which, in turn, triggers the formation of the apoptosome complex including cytochrome c/Apaf-1/caspase-9 and activates caspase-3 [26]. The activation of the caspase cascade decomposes various types of matrix proteins, as well as poly-ADP ribose polymerase (PARP), which resides in the nucleus, to induce apoptosis [27]. Therefore, western blot analysis was performed to detect changes in the expression of Bcl-2 family proteins when DU-145 prostate cancer cells were treated with 500 or 1000 µg/mL fucoidan. Expression of Bax, cleaved caspase-9, and cleaved PARP, all pro-apoptotic proteins, was increased, while that of Bcl-2, an anti-apoptotic protein, was decreased, all in a concentration-dependent manner (Figure 3). A study by Boo et al. [18] also reported a increase in Bax, cleaved caspase-9, and cleaved PARP expression when PC-3 prostate cancer cells were treated with fucoidan.

Park et al. [28] demonstrated that, when T24 colon cancer cells were treated with fucoidan, the expression of Bax and cleaved PARP when increased, whereas Bcl-2 and pro-caspase-9 were downregulated. In summary, fucoidan likely induces apoptosis in cancer cells by regulating the expression of Bax and Bcl-2, inducing cleavage of caspase-9 and PARP.

### 2.4. Effect of Fucoidan on PI3K/Akt Pathways in DU-145 Cancer Cells

**Figure 4:**
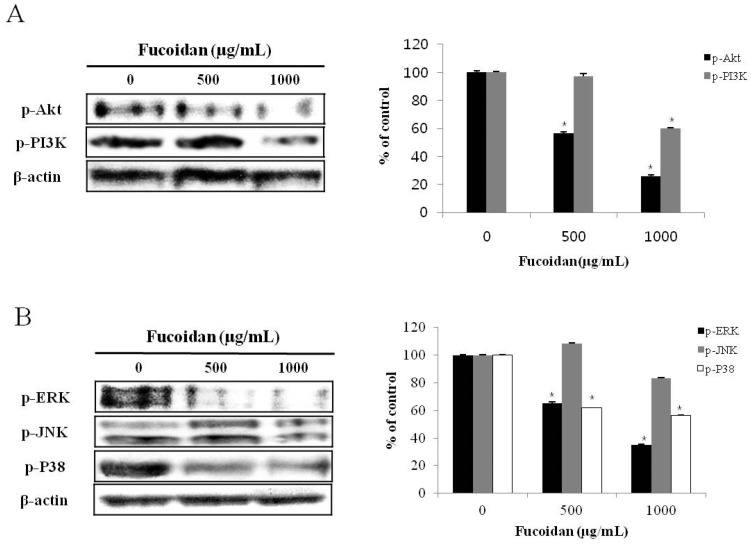
Effect of fucoidan on the activation of PI3K/Akt pathway in DU-145 cells. Cells were treated with fucoidan 0, 500 and 1000 μg/mL for 24 h. Cell lysates were prepared as described in the materials and methods and analyzed by 12% SDS-PAGE followed by western blotting. (**A**) The membranes were incubated with PI3K/AKT pathway antibodies. (**B**) The membranes were incubated with MAPKs pathway antibodies. Each bar represents the mean ± SD calculated from independent experiments. Significance was determined by Dunnett’s *t*-test with * *p* <0.05 compared as statistically significant compared with non-treated controls.

Akt is a serine/threonine kinase, also called protein kinase B, is activated by PI3K and regulates many biological responses, including proliferation, differentiation, or the cell cycle-associated survival of cells. When triggered by PI3K, Akt inhibits apoptosis by hindering the expression of BAD (Bcl-2 associated death promoter) or caspase-9, both of which are pro-apoptotic [29]. To investigate the effect of fucoidan on PI3K/Akt signaling pathways, western blotting was performed (Figure 4A). When DU-145 prostate cancer cells were treated with 500 or 1000 µg/mL fucoidan for 24 h, the phosphorylation of Akt and PI3K was decreased in a concentration-dependent manner. In a similar experiment confirmed the apoptosis of androgen-independent PC-3 human prostate cancer cells [18] also reported a decrease in p-Akt dependent on concentration of fucoidan. A study by Lee et al. [29] also reported a decrease in p-Akt and p-PI3K dependent on time and concentration when A549 lung cancer cells were treated with fucoidan. Together, these studies indicate that fucoidan induces apoptosis in DU-145 prostate cancer cells by decreasing activation of Akt and PI3K.

### 2.5. Effect of Fucoidan on MAPK Pathways in DU-145 Cancer Cells

Various kinases in the MAPK pathways are involved in diverse actions depending on cellular conditions; generally, the ERK signaling pathway is involved in cell proliferation, whereas JNK acts to antagonize cell proliferation [30]. Thus, we investigated how MAPK signaling is affected by fucoidan in DU-145 prostate cancer cells. When DU-145 prostate cancer cells were treated with 500 or 1000 µg/mL fucoidan for 24 h, although there was no change in phosphorylation of JNK, the ERK and p38 was decreased in a concentration-dependent manner (Figure 4B). Boo et al. [18] demonstrated that, when PC-3 prostate cancer cells were treated with fucoidan, the expression of p38 was decreased, whereas p-ERK was upregulated. A study by Aisa et al. [10] also reported a decrease in p-ERK and p-p38 expression when HCT-15 colon cancer cells were treated with fucoidan. These results demonstrate that fucoidan inhibits the growth of DU-145 prostate cancer cells and induces apoptosis by inhibiting the phosphorylation of ERK and p38.

### 2.6. Effect of Fucoidan on Tumor Growth In Vivo Animal Model

**Figure 5:**
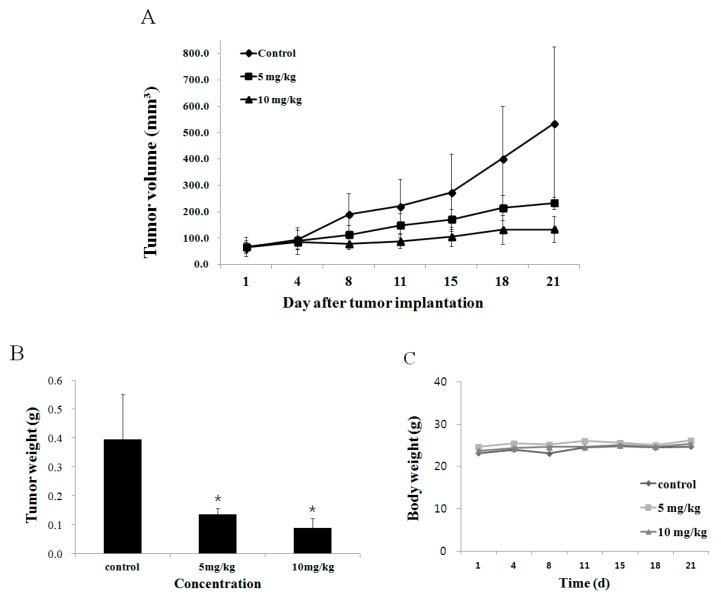
Inhibition of DU-145 prostate tumor growth and enhancement of apoptosis in DU-145 prostate tumors by the fucoidan. (**A**) To identify the effect of fucoidan in DU-145 prostate tumor growth, nude mice were treated with fucoidan (0, 5, 10 mg/kg) for 21 days (*n* = 5). (**B**) The graph expresses final tumor weight. (**C**) The graph is nude mice weight. Each value was expressed as mean ± SE of five mice. Significance was determined by Dunnett’s *t*-test with * *p* <0.05 compared as statistically significant compared with non-treated controls.

Next, we evaluated the effect of fucoidan on tumors arising from transplantation of DU-145 cells into nude mice. The tumor size was measured twice a week, and fucoidan was diluted with phosphate-buffered saline and intraperitoneally injected at 5 or 10 mg/kg body weight five times a week for three weeks. Tumors were smaller in mice given fucoidan compared with the control group eight days from the commencement of drug administration. At 21 days, compared with the control group, tumors in the low-concentration (5 mg/kg) group were 52.0% smaller, while those in the high-concentration (10 mg/kg) group were 80.0% smaller (Figure 5A). In terms of mass, the mean weight of tumors in mice receiving 5 mg/kg fucoidan was 137 ± 19.0 mg and 89 ± 33.0 mg in those receiving 10 mg/kg; there was a marked trend of decreased tumor weight compared with the control group, in which tumors were 397 ± 15.4 mg (Figure 5B). Han et al. [15] reported that intraperitoneal injection of 5 or 10 mg/kg fucoidan into colon cancer (HT29)-bearing mice led to a decrease in the tumor weight by 39.0% ± 2.6% or 7.5% ± 1.2%, respectively. Such results lead to the reasonable conclusion that fucoidan also inhibits the growth of DU-145 prostate tumors.

### 2.7. Effect of Fucoidan on the Apoptosis Induction of DU-145 Tumor Tissue

**Figure 6:**
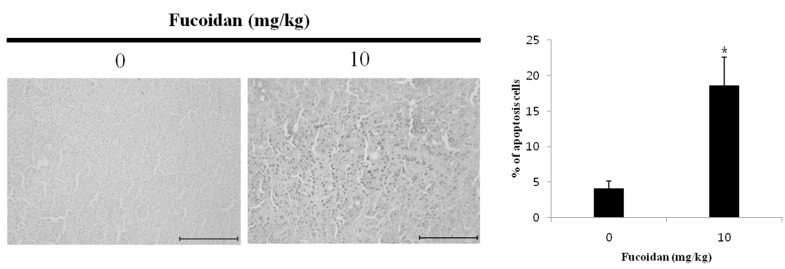
Induction of apoptosis by fucoidan in DU-145 cells. Nude mice were treated with fucoidan for 21 days and apoptosis was assessed by terminal deoxynucleotidyltransferase-mediated Dutp nick-ned labeling (TUNEL) assay. Tumor tissues were observed under a microscope and photographed at a ×200 magnification. The percentage of labeled with TUNEL-positive apoptotic cells was calculated from 1,000 scored cells. Paraffin-embedded tumors were cut into 5 μm sections. Each bar represents the mean ± SD calculated from independent experiments. Significance was determined by Dunnett’s *t*-test with * *p* <0.05 compared as statistically significant compared with non-treated controls. Scale bar, 10 µm.

To verify the anticancer effect of fucoidan on DU-145 prostate cancer, the drug was administered to mice carrying xenograft tumors. Compared with the control group, tumors from mice given 0 mg/kg or 10 mg/kg fucoidan had more apoptotic cells (4.1% ± 1.0% and 18.6% ± 4.0% more, respectively as measured by the TUNEL (terminal deoxynucleotidyl transferase dUTP nick end labeling) assay (Figure 6). This result implies that DNA fragmentation observed earlier resulted from apoptosis. Xue et al. [31] reported similar effects of 5 or 10 mg/kg fucoidan on 4T1 breast cancer xenografts in mice. Thus, fucoidan likely inhibits the growth of tumors by inducing apoptosis in DU-145 prostate cancer cells.

### 2.8. Effect of Fucoidan on Akt and ERK Expression in DU-145 Tumor Tissue

**Figure 7:**
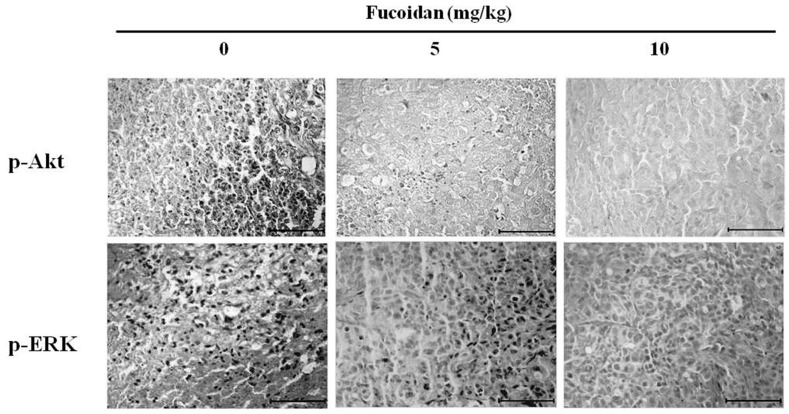
Effect of fucoidan on p-Akt and p-ERK expression in DU-145 prostate tumors. Nude mice were administered fucoidan (0, 5 and 10 mg/kg) for three weeks and assayed by immunohistochemistry using p-Akt and p-ERK antibodes. Tumor tissues were observed under a microscope and photographed at a ×400 magnification. Paraffin-embedded tumors were sectioned to a thickness of 5 µm. Scale bar, 5 µm.

Akt and ERK cooperate to maintain cell viability, and are known to be involved in proliferation and differentiation of cells and to regulate a wide spectrum of biological activity [32]. Thus, we employed immunohistochemical assays to measure the activation (phosphorylation) of Akt and ERK in tumor tissues collected from human tumor-xenografted mice after intraperitoneal injection of 5 or 10 mg/kg fucoidan (Figure 7). We observed that fucoidan at either dose decreased phosphorylation of Akt and ERK compared with the control group, similar to the results of western blotting in vitro. Fucoidan thus appears to inhibit the growth and migration of tumors by regulating the activity of Akt and ERK.

### 2.9. The Histopathological Changes in DU-145 Tumor Tissues by the Fucoidan

**Figure 8:**
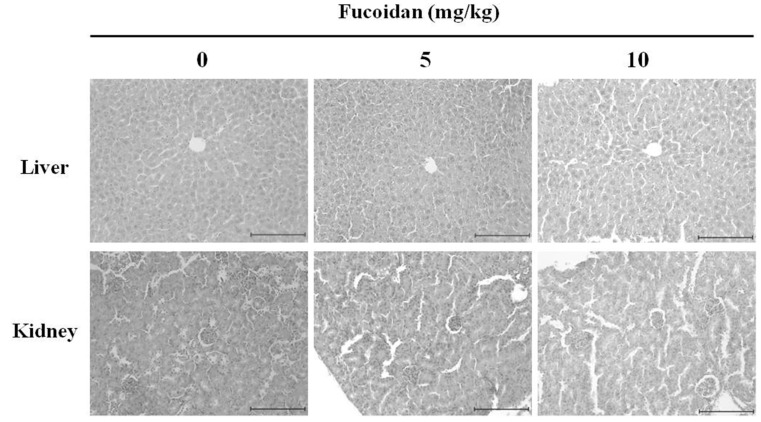
Histological observation of nude mice treated intraperitoneally with fucoidan. Fucoidan was administered at a dose of 5 or 10 mg/kg five times per week, for a total 21 injections. On day 21, mice were sacrificed, and tumors excised and evaluated by hematoxylin & eosin (H & E) staining (×200). The dose of fucoidan had no detectable toxicological effect on nude mice. Scale bar, 10 µm.

Next, to assess organ toxicity due to fucoidan administration, liver, and kidney tissues from tumor-xenografted mice were histologically examined by hematoxylin and eosin staining followed by fluorescence microscopy (original magnification, ×200). No histopathological abnormality was detected, indicating that fucoidan causes no detectable toxic effects (Figure 8).

## 3. Materials and Methods

### 3.1. Chemicals, Drugs, and Antibodies

Fucoidan *(Undaria pinnatifida)* was purchased from Sigma-Aldrich (St. Louis, MO, USA). RPMI-1640 medium, penicillin-streptomycin, trypsin-EDTA and fetal bovine serum (FBS) were purchased from HyClone Laboratories Inc. (Logan, UT, USA). 3-(4,5-Dimethythiazol-2-yl)-2,5-diphenyl tetrazolium bromide (MTT) and dimethylsulfoxide (DMSO) were obtained from Sigma-Aldrich. Antibodies against Bax, Bcl-2, β-actin, Akt, phospho-Akt (Ser473), caspase-9, phospho-PARP, phosphoinositide 3-kinase (PI3K), extracellular signal regulated kinase (ERK) 1/2, c-Jun *N*-terminal kinase (JNK), P38 and rabbit lgG were purchased from Cell Signaling Technology (Beverly, MA, USA). Cell lysis buffer and 4',6-diamidino-2-phenylindole (DAPI) were purchased from Invitrogen Life Technologies (Carlsbad, CA, USA). The DeadEnd™ fluorometric terminal deoxyribonucleotide transferase-mediated dUTP nick end-labeling (TUNEL) assay kit was purchased from Promega (Madison, WI, USA).

### 3.2. Cell Lines and Culture

The human prostate carcinoma cell line, DU-145, was purchased from the Korean Cell Line Bank (Seoul, Korea) and maintained in RPMI-1640 medium supplemented with 5% FBS and 1% penicillin-streptomycin at 37 °C in a humidified 5% CO_2_ atmosphere. Culture medium was renewed every 2–3 days. For fucoidan treatment, DU-145 cells were seeded at a density of approximately 3 × 10^4^ cells/cm^2^ in a 175 cm^2^ flask and were allowed to adhere overnight.

### 3.3. Cell Viability Assay

The anticancer effects of fucoidan were assessed by MTT assay. DU-145 cells were seeded in a 96-well plate at a density of 2 × 10^4^ cells/mL and a volume of 200 μL/well. After 24 h of incubation, the cells were treated with 250, 500, 750, and 1000 µg/mL fucoidan for either 24 h in triplicate. Following treatment, the medium was discarded, followed by the addition of 40 μL of a 5 mg/mL MTT solution and incubation for a further 2 h. The medium was then aspirated and the formazan product generated by viable cells was solubilized with the addition of 100 μL of DMSO. The absorbance of the solutions at 595 nm was determined using a microplate reader (Bio-Rad, Hercules, CA, USA). The percentage of viable cells was estimated in comparison to the untreated control cells.

### 3.4. 4′,6-diamidino-2-phenylindole (DAPI) Staining

Apoptotic cell death was determined morphologically using a fluorescent nuclear dye, DAPI. DAPI staining showed the number of apoptotic cells with chromatin condensation and nuclear fragmentation. DU-145 cells were incubated with PBS or various concentrations of fucoidan (500 and 1000 μg/mL) for 24 h, then harvested by trypsinization, and fixed in 70% ethanol overnight at 4 °C. The next day, the cells were stained with DAPI, deposited onto the slides, and finally viewed to detect apoptotic characteristics with a fluorescent microscope.

### 3.5. Western Blot Analysis

Cells were treated with various concentrations of fucoidan for 24 h, and then protein concentrations were determined using the Bradford protein assay (Bio-Rad Laboratories, Hercules, CA). Total proteins in each cell lysate were resolved on various concentrations (6%–14%) of sodium dodecyl sulfate-polyacrylamide gel electrophoresis (SDS PAGE) gels, and then were electro-transferred onto nitrocellulose membranes. The membranes were incubated with blocking buffer (5% non-fat dry milk in Tris-buffered saline with Tween 20 (TBS-T)) for 1 h at room temperature, and then were further incubated with specific antibodies diluted in blocking solution overnight at 4 °C. After washing with TBS-T, membranes were incubated with horseradish peroxidase (HRP)-conjugated secondary antibodies for 1 h at room temperature. After washing, bands were visualized using enhanced chemiluminescence (ECL) detection reagents (Pierce Biotechnology, Rockford, IL, USA) according to the manufacturer’s instructions.

### 3.6. Antitumor Activity In Vivo

Five-week-old male BALB/c nude mice (nu/nu) were purchased from Orient Bio Inc. (Gyeonggi-do, Korea). Experiments on animals were performed in accordance with the Guidelines for the Care and Use of Animals of the Kongju National University Animals Care Committee (Chungcheongnam-do, Korea). Mice were maintained under a 12 h light/dark cycle, and housed under controlled temperature (23 ± 3 °C) and humidity (40% ± 10%) conditions. Mice were allowed access to laboratory pelleted food and water ad libitum. DU-145 cells were maintained in RPMI-1640 supplemented with 10% FBS and 1% penicillin-streptomycin at 37 °C in a humidified 5% CO_2_ atmosphere. DU-145 cells were harvested from cultures by exposure to 0.25% trypsin. Trypsinization was stopped with a solution containing 10% FBS, and cells were then washed twice and resuspended in RPMI-1640 medium. A total of 2 × 10^7^ cells in 0.2 mL of medium were injected subcutaneously into the right flank of donor nude mice. Seven days after the subcutaneous injection, DU-145 cells growing under the skin of nude mice established tumors. All animal experiments were performed following the approval of the Institutional Animal Care and Use Committee according to the guidelines of Kongju National University (KNU_2015-06). 

### 3.7. Histological Examination

The excised livers and kidneys were immediately fixed in 10% neutral-buffered formalin and, after embedding in paraffin, cut into 5-μm-thick sections. Following hematoxylin and eosin (H & E) staining, the sections were examined under a light microscope (×200).

### 3.8. TUNEL Assay

Paraffin-embedded tumor tissues were used for TUNEL staining, which was performed using the Dead End Colorimetric TUNEL system (Promega). Paraffin-embedded sections (5 μm thick) were processed according to the manufacturer’s protocol. Briefly, sections were deparaffinized in xylene, and then treated with a graded series of alcohol (100%, 95%, 85%, 70%, and 50% ethanol (*v/v*) in double-distilled H_2_O) and rehydrated in PBS (pH 7.5). Then the tissues were treated with proteinase K solution for permeabilization and refixed with 4% paraformaldehyde solution. Slides were treated with the rTdT reaction mix and incubated at 37 for 1 h; reactions were terminated by immersing the slides in 2× SSC solution for 15 min at room temperature. After blocking endogenous peroxidase activity (with 0.3% hydrogen peroxide), slides were washed with PBS, and then incubated with streptavidin horseradish peroxidase solution for 30 min at room temperature. After washing, slides were incubated with 3,3-diaminobenzidine (substrate) solution until a light brown background appeared (10 min), and then rinsed several times in deionized water. After mounting, slides were observed using a light microscope.

### 3.9. Immunohistochemistry

To detect p-Akt and p-ERK, 5-μm-thick sections were cut from paraffin-embedded tissue blocks. The sections were deparaffinized and hydrated by sequential immersion in xylene and graded alcohol solutions. The endogenous peroxidase activity was quenched by treatment with 3% hydrogen peroxide for 5 min at room temperature. The sections were incubated with primary antibodies for 1 h at 37 °C and then with the secondary antibody for 30 min at room temperature. Staining was performed using diaminobenzidine (DAB) and counterstaining was performed using methyl green. For the negative control, the incubated antibody diluent was used as a substitute for the primary antibody

### 3.10. Statistical Analysis

The results are expressed as the means ± standard deviation (SD). Differences between the mean values for the groups were assessed by a one-way analysis of variance (ANOVA) and Dunnett’s *t*-tests. *p* < 0.05 was considered to indicate a statistically significant difference.

## 4. Conclusions

In this study, we confirmed the effects of fucoidan on androgen-independent prostate cancer via in vitro and in vivo. Fucoidan inhibited the proliferation of and induced apoptosis in DU-145 prostate cancer cells and modulated protein expression associated with apoptosis through the PI3K/Akt and MAPK signaling pathways. Administration of 5 or 10 mg/kg fucoidan to DU-145 prostate-bearing nude mice significantly reduced tumor volume and tumor weight. TUNEL assay results suggest that this results from fucoidan’s induction of cancer cell apoptosis. Finally, immunohistochemical analysis of tumor tissue revealed that fucoidan treatment decreased p-Akt and p-ERK levels, indicating that apoptosis of DU-145 prostate cancer cells results from regulation of the PI3K/Akt and MAPK signaling pathways. Thus, the present study provides a molecular basis for the use of fucoidan as a cancer chemopreventive and chemotherapeutic agent.
